# Non-negligible greenhouse gas emissions from non-sewered sanitation systems: A meta-analysis

**DOI:** 10.1016/j.envres.2022.113468

**Published:** 2022-09

**Authors:** Shikun Cheng, Jinyun Long, Barbara Evans, Zhe Zhan, Tianxin Li, Cong Chen, Heinz-Peter Mang, Zifu Li

**Affiliations:** a School of Energy and Environmental Engineering, Beijing Key Laboratory of Resource-oriented Treatment of Industrial Pollutants, University of Science and Technology Beijing, Xueyuan Road No.30, Haidian District, Beijing, 100083, PR China; b School of Civil Engineering, University of Leeds, Leeds, UK; c School of Economics and Management, University of Science and Technology Beijing, Xueyuan Road No.30, Haidian District, Beijing, 100083, PR China; d German Toilet Organization, Paulsenstr. 23/12163, Berlin, Germany

**Keywords:** Non-sewered sanitation systems (NSSS), IPCC accounting Method, GHG emissions, Methane emissions

## Abstract

Current methods for estimating sanitation emissions underestimate the significance of methane emissions from non-sewered sanitation systems (NSSS), which are prevalent in many countries. NSSS play a vital role in the safe management of fecal sludge, accounting for approximately half of all existing sanitation provisions. We analyzed the distribution of global NSSS and used IPCC accounting methods to estimate the total methane emissions profiles from these systems. Then, we examined the literature to establish the level of uncertainty associated with this accounting estimate. The global methane emissions from NSSS in 2020 was estimated to as 377 (22–1003) Mt CO_2_e/year or 4.7% (0.3%–12.5%) of global anthropogenic methane emissions, which are comparable to the greenhouse gas (GHG) emissions from wastewater treatment plants. NSSS is the major option for open defecation and is expected to increase by 55 Mt CO_2_e/year after complete open defecation free. It is time to acknowledge the GHG emissions from the NSSS as a non-negligible source.

## Highlights

• The global distribution of NSSS was partially visualized.
• The CH_4_ emission from global NSSS was tentatively calculated.
• The impact of temperature on the in-situ monitoring was briefly analyzed.
• The carbon emissions from NSSS and WWTPs were generally compared.

## 1 Introduction

The global population in 2020 has reached 7.8 billion and is projected to increase to 8.5 billion by 2030 (United Nations, 2019a). This growing population results in increased production of human feces. Based on the latest empirical data (Rose et al., 2015) amounts to a total global production of human feces of between 1.43 and 22.38 × 10^11^ kg/year (wet weight), and 57% could not be treated in a centralized manner through sewers (WHO. UNICEF., 2021). Although access to sanitation has been steadily climbing with high economic growth and urbanization rates, 13% of people in rural areas still practice open defecation (OD) (WHO. UNICEF., 2021). Sanitation without sewers, including pit latrines and septic tanks, seems a feasible solution for decentralized fecal management. It is particularly prevalent in Central Asia, South Asia, and South Africa and dominate service provision (WHO. UNICEF., 2021). Sanitation without sewer is often poorly managed, and environmental problems are associated with absent or inadequate fecal sludge management (Peal et al., 2020). However, they can be highly effective for public health and the environment if it is well managed.

MIT Technology Review (Winick, 2019) selected sanitation without sewers as one of the top 10 breakthrough technologies in 2019, following the introduction of the international standard *ISO 30500: 2018 Non-sewered sanitation systems (NSSS)* in 2018. The existing NSSS does not fully meet the ISO 30500 standard, which complies with performance of a technology (product standard). The ideal NSSS collects, conveys, and fully treats the specific input within the system to allow the safe reuse or disposal of the generated solid, liquid, and gaseous output, and is not connected to a networked sewer or networked drainage system. ISO 30500 defines the requirements of identified risks, safety, process controls, and other relevant aspects (Cid et al., 2022). In reality, for instance, the septic tanks without a path of fecal disposal have an environmental discharge risk. The biogas-linked toilets are often abandoned because of inadequate feedstock and lack of maintenance, which immediately causes odor emissions problems and cannot usually achieve hygienic effects. The pit latrines and urine-diverting toilets do not even meet the ergonomic design easily (Cheng et al., 2018). According to WHO. UNICEF. (2021), an estimated 1.7 billion people worldwide use septic tanks and 1.6 billion use pit latrines. In septic tank systems, including soakaway or leach fields and septic tanks, a partial treatment process occurs inside the pit through the anaerobic digestion of sludges, with some aerobic decomposition also occurring near the surface. In pit latrines, anaerobic processes may often dominate, with some aerobic decomposition occurring in the top layer of the pit (van Eekert et al., 2019). Anaerobic and aerobic decompositions result in GHG production, such as CO_2_, CH_4_, and N_2_O, of which CH_4_ may often be considered the major contributor to GHG during the anaerobic process. The reason is that CO_2_ is generally considered biogenic (or natural attribution organic matter), and N_2_O production is a trace compared with CO_2_ and CH_4_.

At the recent 26th UN Climate Change Conference of the Parties (COP26), the global community committed to reduce methane emissions globally. However, accurate estimates of the total global methane production from sanitation have not been studied. Some researchers have conducted GHG emission assessments relating to sanitation. For example, Strokal and Kroeze (2014) estimated the N_2_O emissions from global human excreta at 0.24 Mt, of which 80% were not associated with centralized wastewater treatment. Moreover, van Eekert et al. (2019) estimated the CH_4_ emissions from pit latrines globally at 3.8 Mt, accounting for 0.3% of the global carbon emissions. However, these estimates did not consider the distribution of NSSS worldwide. Therefore, this study will focus on the quantitative and geographical distributions of NSSS and present new estimates of the total CH_4_ emissions from the anaerobic digestion of human excreta from global NSSS.

## 2 Materials and methods

### 2.1 Population distribution using NSSS

We aggregated a series of data in 2020 to determine the population of NSSS users by country. Septic tanks, latrine, and other utilization ratios were determined from country-level health and sanitation surveys compiled by the WHO/UNICEF Joint Monitoring Program for Water Supply and Sanitation (WHO. UNICEF., 2021). Detailed data on the nature of pits and tanks underground are unavailable globally. We, therefore, assumed that systems designated as “septic tanks” were sealed tanks or engineered septic tanks. Meanwhile, all other categories (e.g., pit latrine, toilets that flush to pits, ventilated improved pit latrines, pit latrines with concrete slabs, traditional latrines, and pit latrines without slab/open pit) were considered infiltrating pit latrines. The total population of NSSS users in 2020, is estimated by multiplying the NSSS utilization data from the JMP by the total national population for each country as provided by Population Division, United Nations (United Nations, 2019b). Moreover, Arcmap 10.2 software is used to visualize the calculation results, which are conducive to spatial analysis.

### 2.2 CH_4_ emission account method

#### 2.2.1 CH_4_ emission model based on IPCC

The *2006 IPCC Guidelines for National Greenhouse Gas Inventories* suggest a mathematical model (Equation (1)) that can be used to account for CH_4_ emissions from septic tanks and pit latrines (IPCC, 2019). The annual CH_4_ emissions from NSSS for any given country in kg are given by:

(1) CH_4__j_ = P_j_ ∙ BOD_j_ ∙ 0.001∙365 ∙ B_0_ ∙ MCF_i_

where:

j = each country or region;

i = each NSSS category;

P = country population using NSSS in inventory year, [cap];

BOD = country-specific per capita biochemical oxygen demand (BOD) in inventory year, [g/cap/day];

B_0_ = maximum CH_4_ producing capacity with a default value of 0.6 kg CH_4_/kg BOD;

MCF = methane correction factor (fraction).

MCF is based on some limited experimental work carried out primarily in the US. IPCC uses a default value of 0.3 for septic tanks. For latrines, IPCC suggests different MCF values (0.1, 0.5, 0.7) relating to various usage conditions of latrines. Given that specific data on the usage and operation of latrines are unavailable, we have assumed a single average value (0.43) for the MCF value of latrines. Emissions calculations for NSSS in countries based on the IPCC method contain significant uncertainties because of the lack of accurate empirical data on emissions profiles and the condition of NSSS on the ground. IPCC suggests the consideration of uncertainty in the estimates of emission factor (EF) and BOD (IPCC, 2019). The EF of a system depends on its contents’ maximum CH_4_ producing capacity and the fraction in anaerobic conditions. IPCC suggests this leads to an uncertainty of 30% and 50%, respectively. The total BOD at the country level depends on the BOD per capita and the population of NSSS users, of which IPCC considers 30% and 5% uncertainty, respectively.

#### 2.2.2 CH_4_ emission model based on experiments

Based on the fecal anaerobic digestion experiments, the per capita average of CH_4_ production from human excreta can be obtained from Equations (2), (3), (4):

(2) CH_4_ = MP ∙ VS ∙ P ∙7300

(3) VS=W∙VS/TS

(4) 7300 = 16 × 365 × 28/22.4

where:

MP = average methane production, [L CH_4_/g VS];

VS = volatile solids in the substrates, [g VS/cap/d];

W = dry weight of human excreta, the value was a range with 12–81 g VS/cap/d (Rose et al., 2015);

VS/TS = proportion of VS in total solids;

P = total population, [cap];

16 = molar weight of CH_4_, [g/mol];

365 = total days in a year, [d];

28 = measure for the impact with global warming potential of CH_4_ relative to CO_2_;

22.4 = molar gas volume at 0 °C, [L/mol].

#### 2.2.3 N_2_O emission model of OD

The GHG emissions from OD can be obtained from Equation (5):

(5) N_2_O=P ∙ N ∙ EF_N2O_ ∙ 44/28

where:

P = global population of practicing OD, the value is 494 million (WHO. UNICEF., 2021), [cap];

N = annual average N excretion per capita, the average value is 657 (329–1789) g N/cap/year (Rose et al., 2015);

EF_N2O_ = EF for direct N_2_O emissions from OD, the value of N input to the soil is 0.01 (0.001–0.018) g N_2_O–N/g N (IPCC, 2019);

44/28 = conversion of N_2_O–N emissions to N_2_O emissions.

### 2.3 Literature selection and data extraction

Estimating the total methane emissions from human excreta is possible by using the general distribution of broad categories of NSSS. Global estimates of total emissions have been based on a simple emission model from typical NSSS systems. The approach from IPCC can generate an easy-to-understand global estimate that nonetheless may under or overestimate total emissions because it does not consider the actual performance of systems as they are found in situ. The calculation results can be improved by using secondary literature to generate more detailed estimates of the emission profiles from typical NSSS systems.

To improve our understanding of the likely range of critical parameters in the accounting model, we retrieved secondary literature on the Web of Science systematically. The keywords, including anaerobic digestion, blackwater, human feces or excreta, ecological sanitation (ecosan), sustainable sanitation (susan), greenhouse gas, and methane, were searched for relevant papers. Then, they were reviewed based on their title and abstract to select papers that focused on the methane emissions from human feces. Relevant papers were then subjected to a full-text inspection. Data were extracted from papers based on the following conditions: (1) substrate was human excreta and not co-digested with other organic compounds; (2) complete anaerobic digestion environment; (3) methane production was converted into data under standard conditions (273.15 K, 101.325 kPa).

## 3 Results and discussion

### 3.1 Geographical distribution of NSSS

**Fig. 1:**
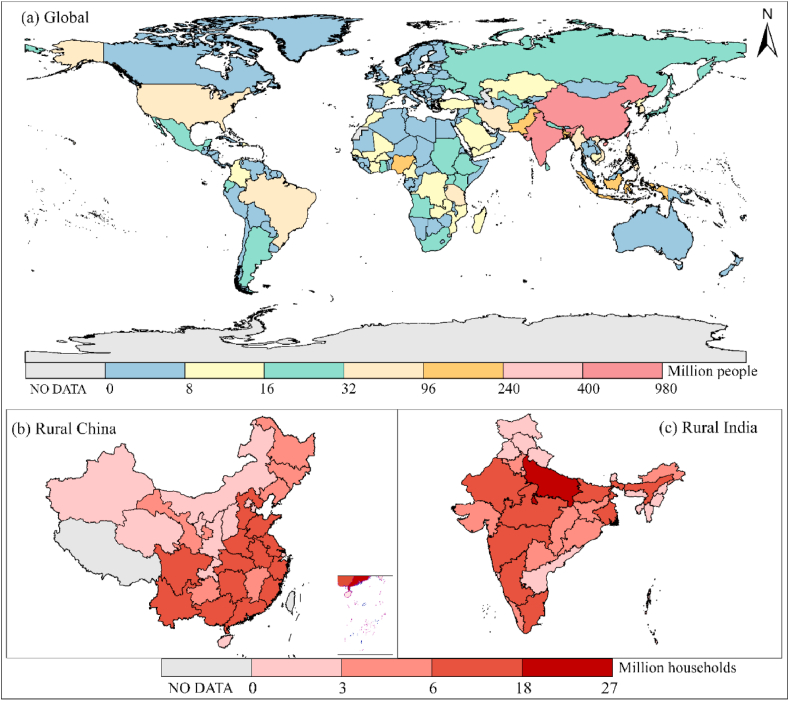
Population of NSSS users in the world (a) and NSSS users in rural China households (b) and rural India households (c). Data sources:(NHC. 2018; National Statistical Office (NSO), 2018; WHO. UNICEF., 2021).

Statistics indicate a potential link between NSSS coverage and the development level of a country. The prevalence of NSSS tends to be lower in more affluent countries; and seven-eighths of high-income countries have NSSS rates below 20%, but most low- and middle-income countries have much higher rates. Fig. 1(a) shows that the most significant populations that use NSSS are those in East, South, and Southeast Asia, particularly in India and China. India, at about 979 million, has the largest population that uses NSSS, followed by China at 400 million, which is more than 10 times higher than other continents (e.g., Europe, Africa, Oceania, and Americas). In urban areas, the rates of connectivity to sewers are higher in China (84%) than in India (34%) (WHO. UNICEF., 2021). NSSS is more widely distributed in rural areas. Fig. 1(b) shows that most households that use NSSS in rural China are in the central, eastern, and southern regions related to population aggregation and reflects the population transition trend to developed regions. By contrast, the distribution of NSSS in rural India is more balanced (Fig. 1(c)). Uttar Pradesh, India has the highest number of households that use NSSS, probably because it is the most populous region in the country. More households use NSSS in rural China than in rural India, and septic tanks, as the central NSSS type, account for 46% and 60%, respectively (National Health Commission, 2018; National Statistical Office (NSO), 2018). In 2020, 494 million people still practiced OD, over a third of whom were from India (WHO. UNICEF., 2021). Sanitation in low-income countries has long been criticized externally. However, NSSS has contributed to ending OD in the past decades. In India, Nepal, Cambodia, and Ethiopia, the population that practices OD dropped by 60% and mainly opted for NSSS as an alternative in 2000–2020 (WHO. UNICEF., 2021; WHO 2019). NSSS may be the most feasible solution to eliminate OD. The ultimate goal of national unity is to achieve open defecation free (ODF) and safe sanitation management. Developing countries, such as India, need to reform sanitation urgently to cope with population pressure, higher health requirements, and climate issues.

### 3.2 The CH_4_ emissions from NSSS based on the IPCC accounting method

**Fig. 2:**
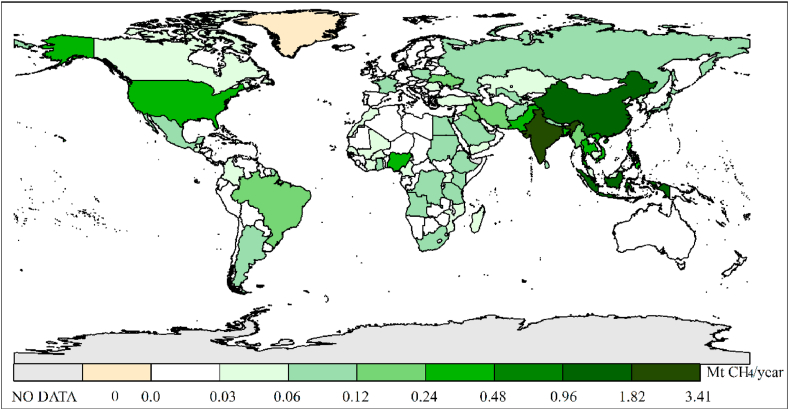
CH_4_ emissions from NSSS (Mt CH_4_/a).

**Fig. 3:**
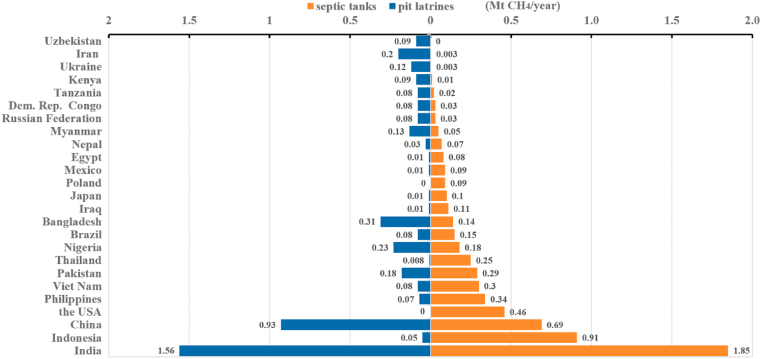
Emissions from septic tanks and pit latrines from the total CH_4_ emissions from NSSS in the top 25 countries.

Based on the IPCC (2019), the annual CH_4_ emissions from all NSSS in 225 countries or regions in 2020 were calculated. In 2014, IPCC updated the global warming potential (GWP) values of CH_4_ and N_2_O to 28 and 265, respectively (IPCC, 2014). These GWP values can be used to convert emissions into CO_2_e, thereby giving a total annual CH_4_ emission estimate for global NSSS of 211 Mt CO_2_e for septic tanks and 166 Mt CO_2_e for pit latrines. The average total CH_4_ emissions from global NSSS is 377 Mt CO_2_e/year with an uncertainty range from (88–1003) Mt CO_2_e/year, which represents 4.7% of the global anthropogenic methane emissions (8047 Mt CO_2_e) (USEPA, 2019). The results are summarized in Fig. 2, which reflect the differences in NSSS methane emissions among countries intuitively. The top five countries with the highest CH_4_ emissions at 95.4, 45.4, 26.9, 13.1, and 12.9 (Mt CO_2_e/year) are India, China, Indonesia, Pakistan, and the USA, respectively (see Fig. 3).

Shaw et al. (2021) also estimated the GHG emissions of NSSS as 560 Mt CO_2_e/year, which is slightly higher than our results. Shaw et al. (2021) reported their results as 20 Mt CO_2_e/year, but these data seemed to have a unit problem not multiplied by the GWP value of CH_4_ relative to CO_2_. We have corrected this problem. Shaw's higher estimates can be attributed to the use of a different assumer EF and population data projected for 2030. A more interesting comparison is with Reid et al. (2014), wherein the distribution of pit latrines globally has changed. India overtook China as the country with the largest in CH_4_ emissions from pit latrines, which increased from 0.32 Mt CH_4_ in 2015 (Reid et al., 2014) to 1.56 Mt CH_4_ in 2020 (this paper), with an average annual increase of more than 37%. The growth is mainly attributable to the very rapid progress made in India to extend onsite sanitation throughout rural and urban areas during the Swatchh Bharat Mission (SBM). A 14% increase was observed in the population accessing toilets in India between 2015 and 2020 (WHO. UNICEF., 2021). During the same period, CH_4_ emissions from pit latrines in China have decreased by 0.17 Mt CH_4_ because the focus of the toilet revolution in China has been to rebuild and improve existing toilets, with less than 1% of the population having no access to the beginning of this period (WHO. UNICEF., 2021). The comparison with Reid et al. (2014) also found that similar progress in extending access to sanitation accounted for increases in CH_4_ emissions in Pakistan, Kenya, the Philippines, South Africa, Ghana, Kazakhstan, and Turkey. Consistent with Reid et al. (2014), African countries are speculated to have a relatively significant growth because of strong population growth and continued dependence on pit latrines in rural and urban areas.

### 3.3 The CH4 emission from human waste based on the literature

With the selection and extraction requirements, 14 publications with 14 groups of data in total were selected (Table 1).

**Table 1:** Fourteen groups of literature data on anaerobic digestion with human feces as substrate.

Substrates	TS^a^ (g/L)	VS^b^(g/L)	VS/TS (%)	MP^c^(L/gVS)	Reference
Fresh feces	67.1	55.3	82.4	0.36	Kim et al. (2019)
Top layer feces in dry toilet	67	52.6	78.5	0.243	Wang et al. (2020)
Blackwater	4.4	3.8	86.4	0.26–0.3	Rajagopal et al. (2013)
Fresh feces	219.5	179	81.5	0.177	van Eekert et al. (2019)
Fresh feces	245	201	82.0	0.271	Riungu et al. (2019)
Feces sludge in septic tanks	12	8.54	71.2	0.299	Chatterjee et al. (2019)
Fresh feces	3.2	2.6	81.3	0.449	Zhang et al. (2019)
Blackwater	4.5	2.83	62.9	0.22	Giwa et al. (2021)
Fresh feces	150	130.5	0.87	0.471	Zhang et al. (2017)
Blackwater	145.6	128	87.9	0.327	Duan et al. (2020)
Fresh feces	47.94	35.48	74.0	0.402	Zuo et al. (2021)
Brown water	3.45	2.85	82.6	0.16	Lavagnolo et al. (2017)
Human excreta	15.5	10.1	65.2	0.122	Sun et al. (2017)
Blackwater	NR	4.5	NR	0.124	Wendland et al. (2007)

Note.

a Total solid (TS).

b Volatile solid (VS).

c Methane production (MP).

A simple meta-analysis was used to estimate the average CH_4_ production from human excreta from these data sources, and we concluded that it lies between 0.122 and 0.471 L CH_4_/gVS. It generally refers to the BMP of human excreta, (i.e., the volume of methane produced by a unit volatile solid in an anaerobic environment). Based on Equations (2), (3), (4), the average per capita CH_4_ emissions from human excreta ranged from 6.7 to 244.8 kg/cap/year. With a global population of approximately 7.8 billion in 2020, 52–1922 Mt CO_2_e/year will be produced if all human excreta worldwide are treated by anaerobic digestion. In 2020, the CH_4_ emissions from 43% of NSSS user population range from 22 to 821 Mt CO_2_e/year. These data are valuable and will lead countries and regions to focus on NSSS and incentive progress up the GHG mitigation and water and sanitation development. However, the gap between maximum and minimum is large. Moreover, an ideal environment is estimated without considering the actual effect factors.

### 3.4 GHG emission from NSSS based on in-situ monitoring

Although 43% of the population rely on NSSS globally, only a limited number of studies have been conducted based on the direct measurements of GHG emissions from NSSS to date. Where measurements have been made, they focus on areas, such as farms and lawns tendentiously. Four major recent studies reported emissions that are directly measured from NSSS, and GHG emissions show very high variability. For example, Huynh et al. (2021) only measured the CH_4_ emission in the first chamber of the septic tanks, ignoring the second and third chambers, thereby leading to significantly lower results than other studies. The CH_4_ emissions from Dubber and Gill (2014) and Truhlar et al. (2016) were 164.5 kg CO_2_e/cap/year and 100 kg CO_2_e/cap/year, which are consistent with the above range of emissions obtained through meta-analysis. Diaz-Valbuena et al. (2011) monitored the emissions of CO_2_, CH_4_, and N_2_O, showing that CH_4_ emissions accounted for 89% of the total or 275 kg CO_2_e/cap/year, which is far beyond the maximum estimated above. The reasons for the variability in the results are unclear but may include climate, population, diet, water consumption, temperature, and differences in the nature of the systems being observed.

NSSS have different structural forms; for example, septic tanks can have two or three chambers, pit latrines can have a single pit or two pits, and feces and urine can be collected separately or mixed. The design, construction, and operation of NSSS affect the emission rate of GHG during fecal sludge decomposition. Some commentators suggested that double-pit latrines, the dominant type of NSSS promoted in India, may be the least environmentally friendly in terms of GHG emissions because of its limited and unclear modeling (Kulak et al., 2017, National Statistical Office (NSO), 2018). The use of source-separated blackwater is also suggested, that is, feces and urine are collected separately at the frontend of NSSS, this approach could alleviate at least 60% of N_2_O emission and 20% of CO_2_ emission compared with the mixed treatment (Badeti et al., 2021). Gao et al. (2019) tested the biochemical methane potential of blackwater from different toilet technologies, showing that the methane production from vacuum toilets was lower than the flushing toilets. This result can be attributed to source-separated blackwater that contains higher concentrations of organic matter and ammonia and has no apparent inhibitory effect on the microbial community.

Temperature is particularly significant. The optimal theoretical temperature for anaerobic digestion is 30°C–38 °C, and the anaerobic digestion rate and gas production will decrease with a temperature lower than 12 °C. Therefore, NSSS in cold regions may emit less GHG than those in warm regions. Truhlar et al. (2019) measured the greenhouse gas emissions from septic tanks for a continuous half-year and showed seasonal and diurnal variation patterns. High CH_4_ fluxes were observed in warm summer, and low fluxes were observed in cold winter. However, CH_4_ flux was lower during the daytime than at night, suggesting that other factors dominated temperature. A similar relationship has occurred in other fields, such as in Hu et al. (2020), who found a positive correlation between CH_4_ emissions and temperature in coastal wetlands through meta-analysis.

In summary, a dearth of reliable empirical evidence for the rates of emissions from existing NSSS systems are found on the ground in various countries. This lack of data severely hampers the ability to reliably estimate the total emissions from NSSS globally.

### 3.5 Comparison of GHG emissions from NSSS and wastewater treatment plants (WWTPs)

Despite the importance of NSSS globally and the relatively low proportion of the population with sanitation systems connected to fully functional WWTPs, the latter has received greater research attention concerning GHG emissions from the sanitation sector. Based on the business-as-usual scenario with emission rates consistent with historical levels, USEPA (2019) estimated that the GHG emissions of wastewater that originated from residential, commercial, and industrial sources were 632 Mt CO_2_e/year in 2020. It is about 1.7 times the same as the average CH_4_ emissions of NSSS. In addition, we collected these GHG emission data from country-level WWTPs and compared them with the CH_4_ emissions from NSSS in this study (Table 2). In China and the USA, GHG emissions from NSSS and WWTPs are almost equal. Calculations and comparisons show that the CO_2_ emissions converted by CH_4_ from NSSS are comparable to the total GHG emissions from WWTPs. The direct carbon emissions (CH_4_ and N_2_O) of WWTPs can be avoided or reduced to a large extent through operation optimization and biogas utilization. Managing CH_4_ emissions from NSSS is more challenging because methane capture at the household level is technically devious. The primary interventions are likely to involve more active management of fecal sludge emptying and transport systems.

**Table 2:** Comparison of GHG emissions between NSSS and WWTPs.

Country	the GHG emissions, Mt CO_2_e/year		
	NSSS (this study)^a^	WWTPs (other studies)^b^	
China	45.4	42.9	Ren et al. (2021)
the USA	12.9	13.4	USEPA (2020)
Greece	0.3	0.9	Koutsou et al. (2018)
Canada	1.1	0.7	Sahely et al. (2006)
Argentine	2.2	3.6	Santalla et al. (2013)
Vietnam	10.6	17.1	Hoa and Matsuoka (2015)
Nigeria	11.4	21.3	UNFCCC (2021a)
Poland	2.5	3.6	UNFCCC (2021b)

a The GHG from NSSS does not include CO_2_ and N_2_O, that is, only CH_4_ emission is considered.

b The GHG from WWTPs includes CH_4_ and N_2_O, but CO_2_.

### 3.6 Comparison of GHG emissions from NSSS and OD

Human waste is generally classified as domestic wastewater. The excreta produced by OD is not within the scope of fecal sludge management and is usually degraded by the natural environment, such as soil. Calculated by Equation (5), the 494 million people who practice OD in 2020 would emit 5121 t N_2_O, totaling 1.6 Mt CO_2_e with an uncertainty range of (0.07–6.7) Mt CO_2_e. In terms of the total amount, N_2_O emissions were reduced by 98% compared with the 0.3 Mt N_2_O in 2000 (Strokal and Kroeze, 2014). In per capita emissions, 2.7 kg CO_2_e/cap/year from OD is much smaller than NSSS (this paper, 114 kg CO_2_e/cap/year). NSSS replacing OD is an inevitable trend of social development. It improves people's sanitation level and protects people's health. At the same time, people must bear the negative impact of sanitation development. Every person in the world who abandons OD and uses NSSS would add about 111 kg CO_2_e/year, which means an increase of 55 Mt CO_2_e/year when OD is completely eliminated. Evidence such as these has important implications for the development of sanitation systems, which, as Shaw et al. (2021) argued, will provide a clear path to the sustainable development of NSSS.

## 4 Conclusion

Due to the scattered distribution and difficulty in systematic management of NSSS, the impact of greenhouse gases emitted by NSSS on the environment has been relatively ignored in the research and policy community. However, the contribution of NSSS to global CH_4_ emissions may be significant at around 377 Mt CO_2_e/year, accounting for 4.7% of the total anthropogenic CH_4_ emissions. India and China contribute extensively to methane emissions of NSSS because of their large populations and NSSS utilization. These estimates only cover those emissions related to fecal sludges stored in situ in NSSS. As seen, significant additional work is needed for modifying the GHG emission model of IPCC to generate reliable national estimates for the critical performance parameters. In-situ monitoring would be essential in understanding the actual fluctuations of GHG emissions from NSSS. The limiting factors include the excrement's physical and chemical properties and environmental parameters, such as temperature, moisture content, organic content, and process parameters (e.g., retention time, and shortcut flow). The lack of reliable empirical data explained above results in need for further modification of our initial overall estimate of total emissions from NSSS, which now has an uncertainty range of CH_4_ 22–1003 Mt CO_2_e/year from NSSS.

Up-to-date empirical data on the emissions from in-situ NSSS found on the ground are lacking, thereby resulting in high levels of uncertainty to IPCC estimates. This uncertainty is a particular challenge for countries that wish to include NSSS in their nationally determined contributions. Although NSSS will continue to play an essential role in achieving safely managed sanitation services and ODF worldwide, building up more substantial evidence for the scale of its impact on methane emission is critical. A better evidence base for the drivers of emissions from NSSS would enable a more effective policy and design and implement interventions to mitigate their impact.

## Funding

This work was supported by 10.13039/501100012166National Key Research and Development Plan [2018YFC1903206], Interdisciplinary Research Project for Young Teachers of 10.13039/501100008778USTB [FRF-IDRY-20-012], the Youth Teacher International Exchange & Growth Program of 10.13039/501100008778USTB [QNXM20210029], The 10.13039/100000865Bill and Melinda Gates Foundation [Grant number OPP 1,153,400].

## Declaration of competing interest

The authors declare that they have no known competing financial interests or personal relationships that could have appeared to influence the work reported in this paper.

## References

[bib1] Badeti U., Pathak N.K., Volpin F., Dorji U., Freguia S., Shon H.K., Phuntsho S. (2021). Impact of source-separation of urine on effluent quality, energy consumption and greenhouse gas emissions of a decentralized wastewater treatment plant. Process Sag. Environ..

[bib2] Chatterjee P., Ghangrekar M.M., Rao S. (2019). Biogas production from partially digested septic tank sludge and its kinetics. Waste Biomass Valori.

[bib3] Cheng S., Li Z., Uddin S.M.N., Mang H.-P., Zhou X., Zhang J., Zheng L., Zhang L. (2018). Toilet revolution in China. J. Environ. Manag..

[bib4] Cid A.C., Abiola F., Starkl M. (2022). Can international nonsewered sanitation standards help solve the global sanitation crisis?. Environ. Sci. Technol..

[bib5] Diaz-Valbuena L.R., Leverenz H.L., Cappa C.D., Tchobanoglous G., Horwath W.R., Darby J.L. (2011). Methane, carbon dioxide, and nitrous oxide emissions from septic tank systems. Environ. Sci. Technol..

[bib6] Duan N., Zhang D.J., Khoshnevisan B., Kougias P.G., Treu L., Liu Z.D., Lin C., Liu H.B., Zhang Y.H., Angelidaki I. (2020). Human waste anaerobic digestion as a promising low-carbon strategy: operating performance, microbial dynamics and environmental footprint. J. Clean. Prod..

[bib7] Dubber D., Gill L. (2014). Application of on-site wastewater treatment in Ireland and perspectives on its sustainability. Sustainability.

[bib8] Gao M.J., Zhang L., Florentino A.P., Liu Y. (2019). Performance of anaerobic treatment of blackwater collected from different toilet flushing systems: can we achieve both energy recovery and water conservation?. J. Hazard Mater..

[bib9] Giwa A.S., Zhang X.Q., Memon A.G., Ali N. (2021). Co-digestion of household black water with kitchen waste for a sustainable decentralized waste management: biochemical methane potential and mixing ratios effects. Environ. Eng. Sci..

[bib10] Hoa N.T., Matsuoka Y. (2015). The analysis of greenhouse gas emissions/reductions in waste sector in Vietnam. Mitig. Adapt. Strat. Gl..

[bib11] Hu M.J., Sardans J., Yang X.Y., Peñuelas J., Tong C. (2020). Patterns and environmental drivers of greenhouse gas fluxes in the coastal wetlands of China: a systematic review and synthesis. Environ. Res..

[bib12] Huynh L.T., Harada H., Fujii S., Nguyen L.P.H., Hoang T.T., Huynh H.T. (2021). Greenhouse gas emissions from blackwater septic systems. Environ. Sci. Technol..

[bib13] IPCC (2014).

[bib14] IPCC (2019).

[bib15] Kim J., Kim J., Lee C. (2019). Anaerobic co-digestion of food waste, human feces, and toilet paper: methane potential and synergistic effect. Fuel.

[bib16] Koutsou O.P., Gatidou G., Stasinakis A.S. (2018). Domestic wastewater management in Greece: greenhouse gas emissions estimation at country scale. J. Clean. Prod..

[bib17] Kulak M., Shah N., Sawant N., Unger N., King H. (2017). Technology choices in scaling up sanitation can significantly affect greenhouse gas emissions and the fertiliser gap in India. J. Water, Sanit. Hyg. Dev..

[bib18] Lavagnolo M.C., Girotto F., Hirata O., Cossu R. (2017). Lab-scale co-digestion of kitchen waste and brown water for a preliminary performance evaluation of a decentralized waste and wastewater management. Waste Manag..

[bib19] National Health Commission (NHC) (2018).

[bib20] National Statistical Office (NSO) (2018).

[bib21] Peal A., Evans B., Ahilan S., Ban R., Blackett I., Hawkins P., Schoebitz L., Scott R., Sleigh A., Strande L., Veses O. (2020). Estimating safely managed sanitation in urban areas; lessons learned from a global implementation of excreta-flow diagrams. Front. Environ. Sci..

[bib22] Rajagopal R., Lim J.W., Mao Y., Chen C.L., Wang J.Y. (2013). Anaerobic co-digestion of source segregated brown water (feces-without-urine) and food waste: for Singapore context. Sci. Total Environ..

[bib23] Reid M.C., Guan K., Wagner F., Mauzerall D.L. (2014). Global methane emissions from pit latrines. Environ. Sci. Technol..

[bib24] Ren J.X., Gao Q.X., Chen H.T., Meng D., Zhang Y., Ma Z.Y., Liu Q., Tang J.J. (2021). Simulation research on greenhouse gas emissions from wastewater treatment plants under the vision of carbon neutrality. Clim. Chang. Res..

[bib25] Riungu J., Ronteltap M., van Lier J.B. (2019). Anaerobic stabilisation of urine diverting dehydrating toilet faeces (UDDT-F) in urban poor settlements: biochemical energy recovery. J. Water, Sanit. Hyg. Dev..

[bib26] Rose C., Parker A., Jefferson B., Cartmell E. (2015). The characterization of feces and urine: a review of the literature to inform advanced treatment technology. Crit. Rev. Environ. Sci. Technol..

[bib27] Sahely H.R., MacLean H.L., Monteith H.D., Bagley D.M. (2006). Comparison of on-site and upstream greenhouse gas emissions from Canadian municipal wastewater treatment facilities. J. Environ. Eng. Sci..

[bib28] Santalla E., Cordoba V., Blanco G. (2013). Greenhouse gas emissions from the waste sector in Argentina in business-as-usual and mitigation scenarios. J. Air Waste Manag. Assoc..

[bib29] Shaw K., Kennedy C., Dorea C.C. (2021). Non-sewered sanitation systems' global greenhouse gas emissions: balancing sustainable development goal tradeoffs to end open defecation. Sustainability.

[bib30] Strokal M., Kroeze C. (2014). Nitrous oxide (N2O) emissions from human waste in 1970–2050. Curr. Opin. Environ. Sustain..

[bib31] Sun Z.Y., Liu K., Tan L., Tang Y.Q., Kida K. (2017). Development of an efficient anaerobic co-digestion process for garbage, excreta, and septic tank sludge to create a resource recycling-oriented society. Waste Manag..

[bib32] Truhlar A.M., Rahm B.G., Brooks R.A., Nadeau S.A., Makarsky E.T., Walter M.T. (2016). Greenhouse gas emissions from septic systems in New York state. J. Environ. Qual..

[bib33] Truhlar A.M., Ortega K.L., Walter M.T. (2019). Seasonal and diel variation in greenhouse gas emissions from septic system leach fields. Int. J. Environ. Sci. Technol..

[bib34] UNFCCC (2021). Poland’S national inventory report 2021. https://unfccc.int/documents/274762.

[bib35] United Nations (2019).

[bib36] United Nations (2019).

[bib37] United Nations Framework Convention on Climate Change(UNFCC) (2021). First national inventory report (NIR1) of the federal republic of Nigeria. https://unfccc.int/documents/307093.

[bib38] United States Environmental Protection Agency (USEPA) (2019).

[bib39] USEPA (2020).

[bib40] van Eekert M.H.A., Gibson W.T., Torondel B., Abilahi F., Liseki B., Schuman E., Sumpter C., Ensink J.H.J. (2019). Anaerobic digestion is the dominant pathway for pit latrine decomposition and is limited by intrinsic factors. Water Sci. Technol..

[bib41] Wang H.H., Li Z.F., Zhou X.Q., Wang X.M., Zuo S.Q. (2020). Anaerobic Co-digestion of kitchen waste and blackwater for different practical application scenarios in decentralized scale: from wastes to energy recovery. Water.

[bib42] Wendland C., Deegener S., Behrendt J., Toshev P., Otterpohl R. (2007). Anaerobic digestion of blackwater from vacuum toilets and kitchen refuse in a continuous stirred tank reactor (CSTR). Water Sci. Technol..

[bib48] WHO, UNICEF (2019). Special focus on inequalities.

[bib43] WHO. UNICEF (2021).

[bib44] Winick E. (2019). Sanitation without sewers. Mit. Technol. Rev..

[bib45] Zhang D.J., Duan N., Lin C., Zhang Y.L., Xu Q.Z., Liu Z.D. (2017). Empirical analysis of mass flow and operation performance of a full-scale biogas plant for human feces treatment. Int. J. Agric. Biol. Eng..

[bib46] Zhang L., Guo B., Zhang Q., Florentino A., Xu R., Zhang Y., Liu Y. (2019). Co-digestion of blackwater with kitchen organic waste: effects of mixing ratios and insights into microbial community. J. Clean. Prod..

[bib47] Zuo S., Zhou X., Li Z., Wang X., Yu L. (2021). Investigation on recycling dry toilet generated blackwater by anaerobic digestion: from energy recovery to sanitation. Sustainability.

